# Cardiac myoglobin deficit has evolved repeatedly in teleost fishes

**DOI:** 10.1098/rsbl.2014.0225

**Published:** 2014-06

**Authors:** Daniel J. Macqueen, Daniel Garcia de la serrana, Ian A. Johnston

**Affiliations:** 1 Institute of Biological and Environmental Sciences, University of Aberdeen, Tillydrone Avenue, Aberdeen AB24 2TZ, UK; 2 Scottish Oceans Institute, University of St Andrews, St Andrews, Fife KY16 8LB, UK

**Keywords:** myoglobin, oxygen supply, fish evolution, climate change

## Abstract

Myoglobin (Mb) is the classic vertebrate oxygen-binding protein present in aerobic striated muscles. It functions principally in oxygen delivery and provides muscle with its characteristic red colour. Members of the Antarctic icefish family (Channichthyidae) are widely thought to be extraordinary for lacking cardiac *Mb* expression, a fact that has been attributed to their low metabolic rate and unusual evolutionary history. Here, we report that cardiac Mb deficit, associated with pale heart colour, has evolved repeatedly during teleost evolution. This trait affects both gill- and air-breathing species from temperate to tropical habitats across a full range of salinities. Cardiac Mb deficit results from total pseudogenization in three-spined stickleback and is associated with a massive reduction in mRNA level in two species that evidently retain functional Mb. The results suggest that near or complete absence of Mb-assisted oxygen delivery to heart muscle is a common facet of teleost biodiversity, even affecting lineages with notable oxygen demands. We suggest that Mb deficit may affect how different teleost species deal with increased tissue oxygen demands arising under climate change.

## 1. Introduction

Myoglobin (Mb) is an oxygen-binding haemprotein of the globin family, typically expressed at high levels in aerobic striated muscle [1,2]. ‘Classic’ functions include the storage of oxygen in the intracellular compartment and the enhancement of oxygen diffusion from blood to mitochondria [1,2]. More recently characterized functions in a range of cell-types include the regulation of intracellular nitric oxide and reactive oxygen species (reviewed in [2,3]). High Mb is positively associated with lifestyles or environments that demand efficient oxygen delivery. For example, high levels of Mb are present in the muscles of diving mammals and birds, supporting active foraging behaviour while breath-holding [4].

Conversely, selection on high Mb levels may be relaxed when demands for oxygen delivery are low. An extreme example is provided by three icefish lineages that independently lost Mb expression in striated muscle, following the earlier loss of haemoglobin (Hb) in their common ancestor (reviewed in [5]). All icefishes have low oxygen demands and evolved in habitats where oxygen has been constantly saturated [5]. Such features, by relaxing the need for efficient oxygen transport, were proposed to explain how these losses were sub-lethal [5]. Nevertheless, there is evidence that Mb and Hb deficit is maladaptive, leading to the suggestion that a major lack of competition in icefish habitats was central to the evolutionary persistence of these apparently exceptional traits [5].

One little-cited study suggested that cardiac Mb deficit extends to members of four further teleost families found in temperate latitudes and that are also relatively inactive [6]. Accordingly, we hypothesized that Mb deficit may be more common than widely realized in teleost fishes. We thus characterized the evolution of cardiac Mb expression in species spanning the teleost phylogeny, occupying a broad range of environments and lifestyles.

## 2. Material and methods

Complete material and methods are provided in the electronic supplementary material. Heart phenotypes were established in 22 Actinopterygian species held under normoxia. Total RNA was extracted from 16 species and used as a template for first-strand cDNA synthesis (electronic supplementary material, table S1). cDNAs for each species were used in PCR reactions employing two degenerate primer pairs, the first highly conserved across Teleostei and the second highly conserved across Acanthopterygii (electronic supplementary material, table S2). *Mb* was sequenced in nine species as described elsewhere [7] (Data accessibility section). Quantitative PCR using species-specific primers (electronic supplementary material, table S2) was used to quantify *Mb* mRNA level in 11 teleost species. BLAST was performed against NCBI (http://www.ncbi.nlm.nih.gov/) and Ensembl (http://www.ensembl.org) databases. Non-synonymous (*d*_N_) and synonymous substitution (*d*_S_) rates were estimated using PAML [8]. One-way ANOVA was used to compare *Mb* mRNA levels across species using Minitab v. 16 (Minitab Inc.).

## 3. Results

### (a) Mb deficit has evolved repeatedly in teleosts

**Figure 1.**
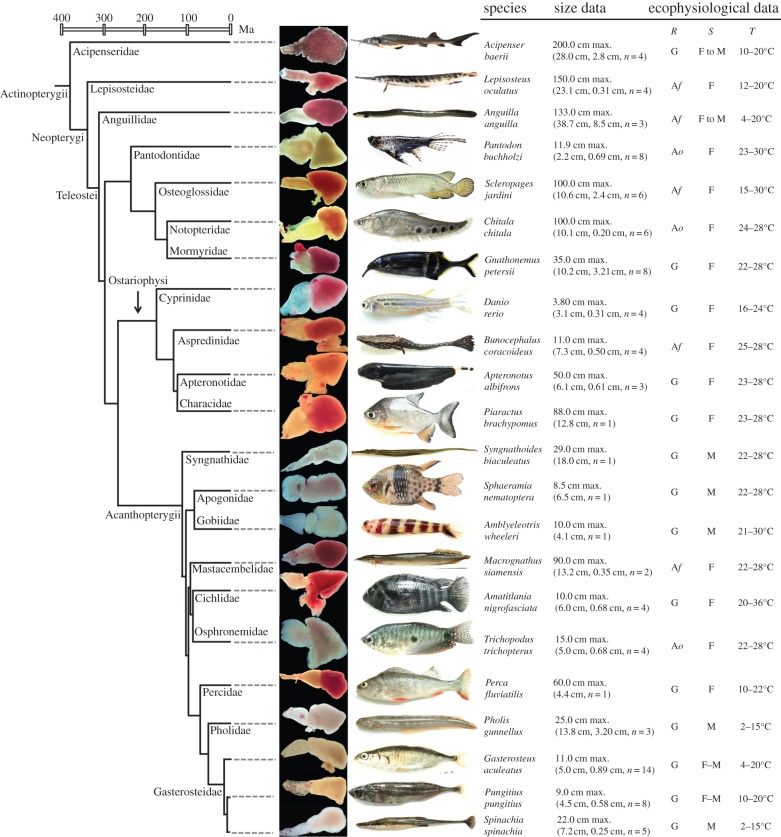
Diversity of cardiac phenotypes in the ray-finned fishes studied, mapped onto a robust phylogeny and timescale [9]. For each species, size data are provided including maximum reported body length (source: FishBase, http://www.fishbase.org/) and body length for the sampled individuals (in parentheses: mean, s.d. and *n*). This latter data show that the range of body sizes sampled was largely randomized across species with respect to heart colour phenotypes. We also provide ecophysiological data on primary respiration phenotype (*R*) (G, gill breather; A*f*, facultative air-breather; A*o*, obligate air-breather), habitat salinity (*S*) (F, freshwater; M, marine; F–M, diadromy possible) and habitat thermal range (*T*). Habitat data were sourced from FishBase and data on air-breathing were acquired from the literature [10].

We mapped heart phenotypes from 22 Actinopterygians onto a robust phylogeny (figure 1). Red pigmentation, indicative of Mb, was present in two species branching before teleosts (figure 1). In teleosts, heart colour ranged from red to pale white/yellow, the latter indicative of Mb deficit [5,6]. Pale hearts have arisen in Osteoglossiformes and on independent occasions in Acanthopterygii, being present in temperate and tropical species from fresh and saltwater (figure 1). All tested species of Gasterosteidae were pale-hearted, as was a closely related species from the Pholidae family (figure 1). Pale-hearted *Syngnathoides biacufeatus* is more closely related to Scombridae [9] than other species in figure 1. As scombrids are active migratory fishes with high Mb, this suggests the existence of a further independent origin for Mb deficit. Pale hearts were present both in species that respire primarily through the gills and via accessory air-breathing organs (figure 1).

### (b) *Mb* PCRs

PCRs targeting cardiac *Mb* cDNAs with two degenerate primer pairs (within the coding region) were successful in nine tested red-hearted species but only two of seven tested pale-hearted species (*Pantodon buchholzi and Trichopodus trichopterus*) (electronic supplementary material, table S1). PCR was successful in species occupying basal clades that are less related to the species that informed primer design than the five pale-hearted Acanthopterygii species [9], where both PCR assays failed. This suggests that *Mb* mRNA may be absent or extremely low in these pale-hearted Acanthopterygii members. Alternatively, normally conserved regions of *Mb* where the primers are binding in other species may not exist, which would be expected if selective pressure to maintain the *Mb* protein-coding sequence had been relaxed during evolution.

### (c) Maintenance of Mb function in pale-hearted species

We established *d*_N_/*d*_S_ ratios comparing the *Mb*-coding region of species pairs within Osteoglossiformes and Acanthopterygii. When *d*_N_/*d*_S_ is less than 1, there is evidence that purifying selection has been a predominant force during evolution, acting to remove changes in amino acid sequence and maintain protein function. Comparison of pale-hearted *P. buchholzi* with three red-hearted osteoglossiform species returned *d*_N_/*d*_S_ ratios of 0.08, 0.18 and 0.24. Comparing solely the red-hearted osteoglossiform species returned similar values (*d*_N_/*d*_S_ = 0.01, 0.17 and 0.22). Comparisons involving four Acanthopterygii species returned *d*_N_/*d*_S_ ratios of 0.09, 0.08 and 0.10 when including pale-hearted *T. trichopterus* (and 0.10, 0.17 and 0.09 excluding this species). These invariant low *d*_N_/*d*_S_ ratios suggest that Mb functions have been maintained as strongly during the evolution of the two pale-hearted species as for their red-hearted relatives.

### (d) *Mb* pseudogenization in stickleback *Gasterosteus aculeatus*

**Figure 2.**
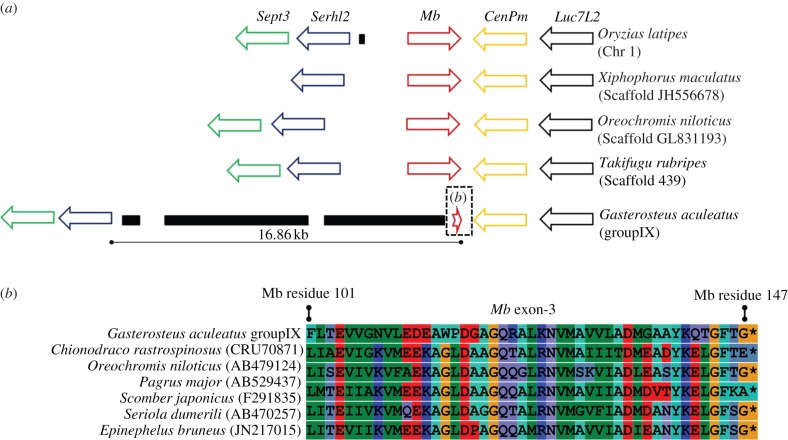
Evidence of *Mb* pseudogenization in three-spined stickleback. (*a*) Genomic neighbourhood surrounding *Mb* of Acanthopterygii members. Orthologous genes are shown as arrows of the same colour. The distance separating *Mb* and *Serhl2* is to scale. Black rectangles show repetitive elements widely distributed in the genome. (*b*) Alignment of Acanthopterygian Mb proteins coded in exon-3 including the stickleback pseudogene ORF. NCBI accession numbers are provided.

It was previously noted that an *Mb* gene was absent from the three-spined stickleback genome assembly [11]. We expanded this observation to better understand the pale stickleback heart. BLAST searches of Mb proteins against the *G. aculeatus* genome produced significant hits on a syntenic region of groupIX containing *Mb* in other Acanthopterygians (figure 2*a*). One of the *G. aculeatus* hits is an open reading frame (ORF) having around 50% protein-level identity to the complete exon-3 of other Acanthoptergians, which includes the Mb stop codon (figure 2*b*).

For most Acanthopterygians, 4.1–5 kb separates *Mb* exon-3 from the last exon of the upstream *Serhl2* gene (figure 2*a*), a region containing *Mb* exon-1 and -2, intron-1 and -2 and the proximal promoter. In stickleback, this region is 16.7 kb long (figure 2*a*) and highly repetitive in the genome: the top 100 BLAST hits (all 0e + 0) for this region are located across 16 additional chromosomes and range in length from 1.3 to 6.3 kb, sharing an average of 98.6% nucleotide identity with the groupIX sequence. The equivalent region in other tested Acanthopterygii species was completely or near-completely non-repetitive within each genome. BLAST searches of assembled *G. aculeatus* traces (NCBI Archive) demonstrated that the groupIX region is covered by multiple overlapping traces, including single traces linking exon-3 to repetitive sequences. The stickleback groupIX region also contains large ORFs that code for conserved proteins with retrovirus domains (respective top BLASTp hits: 1 × 10^−54^ and 0.0). These data suggest that repetitive elements have invaded the region containing the stickleback *Mb* gene, consistent with its pseudogenization.

### (e) Cardiac *Mb* mRNA levels in a range of teleosts

There was extensive variation in cardiac *Mb* mRNA levels across teleost species (table 1; *F* = 84.1, *p* < 0.0001: one-way ANOVA) with red hearts having higher levels than pale hearts. *Pantodon buchholzi* had approximately 250–700 times less mean cardiac *Mb* mRNA than Osteoglossiformes relatives, whereas *T. trichopterus* had approximately 40 times less than its cichlid relative *Amatitlania nigrofasciata* (table 1). These differences were highly statistically relevant (Tukey's test). A cardiac mRNA was transcribed from exon-3 of the *G. aculeatus Mb* pseudogene. However, its significance is contextualized by the lack of potential for translation of a functional Mb protein and its approximate 300- to 500 000-fold lower mean abundance versus the other species (table 1).

**Table 1.** Cardiac *Mb* mRNA levels in 11 teleost species. (Phylogenetic relationships are shown in figure 1.)

species	family	order	heart colour	*Mb* mRNA (mean)	*Mb* mRNA (s.d.)	*n*
*Anguilla anguilla*	Anguillidae	Anguilliformes	red	33.30	11.39	3
*Gnathonemus petersii*	Mormyridae	Osteoglossiformes	red	13.58	2.25	4
*Chitala chitala*	Notopteridae	Osteoglossiformes	red	4.75	1.07	4
*Pantodon buchholzi*	Pantodontidae	Osteoglossiformes	pale^a^	0.02	0.01	4
*Danio rerio*	Cyprinidae	Cypriniformes	red	10.45	1.16	3
*Bunocephalus coracoideus*	Aspredinidae	Siluriformes	red	18.02	0.59	3
*Apteronotus albifrons*	Apteronotidae	Gymnotiformes	orange	17.05	5.97	3
*Salmo salar*	Salmonidae	Salmoniformes	red	5.16	1.43	4
*Amatitlania nigrofasciata*	Cichlidae	Perciformes	red	16.10	2.58	4
*Trichopodus trichopterus*	Osphronemidae	Perciformes	pale^a^	0.41	0.39	4
*Gasterosteus aculeatus*	Gasterosteidae	Perciformes	pale^b^	6.46 × 10^−5^	4.33 × 10^−5^	4

^a^Retains a functional Mb protein.

^b^*Mb* pseudogene.

## 4. Discussion

It is often stated that *Mb* expression is essential for aerobic function in vertebrate striated muscle [1]. However, even before this study, exceptions to this ‘rule’ were suspected, including the entire class Amphibia and a few teleost species [2,5,6]. Nevertheless, such cases have been deemed extraordinary [2,5]. Surprisingly, we revealed that cardiac Mb deficit has evolved repeatedly in teleosts under diverse ecological settings. The pale hearts observed are unlikely to have high compensatory levels of other globin proteins, as posited for amphibians [2], as this would produce red pigmentation.

As classic Mb functions require high expression in myocytes [1], we conclude that oxygen diffusion is happening with little or no assistance from Mb in pale teleost hearts. This is noteworthy because tropical fishes typically require six times more oxygen at rest than polar species such as icefishes [12]. The total loss of Mb function in three-spined stickleback is also paradoxical, as this species can migrate long distances [13]. Under the current paradigm, such ability for aerobic performance should have favoured maintenance of Mb.

Our data also raise the possibility that Mb deficit is common in teleosts as a group. In fact, there is evidence suggesting that Mb deficit may be an ancestral character shared by five Acanthopterygii families containing hundreds of species. All species tested to date from Gasterosteidae, Pholidae, Anarhichadidae, Cyclopteridae and Zoarcidae have pale hearts (this work, [6]) and are more closely related to one another than to the next red-hearted species [9] (*Perca fluviatilis* in figure 1). The massive invasion of repetitive elements into the *Mb* gene region of stickleback (Gasterosteidae) is consistent with an ancient origin for pseudogenization, which provides a hypothesis to explain the pale-heartedness shared by these Acanthopterygii families.

The diversity of species with cardiac Mb deficit implies that a spectrum of biological settings exist where selective pressure on Mb-assisted oxygen supply into heart is relaxed. However, the associated physiological and ecological factors remain uncharacterized. Our data offer limited clues in the way of explanation. First, all identified tropical and temperate species with pale hearts have relatively small adult body size (figure 1), suggesting that some aspect of allometry may affect constraints on Mb-assisted oxygen-transport into hearts. However, as other small species have high *Mb* expression e.g. *Danio* (figure 1 and table 1), additional factors must also be at play. Second, two small obligate air-breathing species have cardiac Mb deficit. This is notable as air-breathing may enhance oxygen supply to the heart [10], perhaps relaxing the need for Mb-assisted oxygen-transport in certain settings, for example in combination with small body size. Overall, the circumstances under which Mb deficit is tolerated are likely to involve many interacting factors—this is an area that demands further attention.

It will also be important to establish whether Mb deficit has potential to negatively affect fitness, as proposed in icefish [5], especially in the context of contemporary climate change. The temperature of the Earth's habitats will rise significantly in the near future [14], increasing the routine oxygen demands of most ectotherms [15]. The ability to meet cellular oxygen requirements dictates the extent of higher physiological functions possible, e.g. behaviour/reproduction [15]. It is hypothesized that when temperature surpasses an ectotherm's optimum range, the capacity to supply enough oxygen to tissues is exceeded, leading to greatly reduced fitness [15,16]. Evidence favouring this model exists for the eelpout *Zoarces viviparous*, where populations may already be suffering under contemporary global warming [16]. Considering Mb's role in supporting cardiac performance, it is plausible that Mb deficit will affect the ability to meet tissue oxygen demands in warmer future habitats. Intriguingly, *Z. viviparous* is part of the aforementioned Acanthopterygii group that may share Mb deficit.

## Supplementary Material

Materials and methods

## Supplementary Material

Table S1

## Supplementary Material

Table S2

## Notes

All experiments were approved by the University of St Andrews Animal Ethics and Welfare Committee.

## Data accessibility

Accession numbers for new *Mb* cDNAs: KJ561849–KJ561856. More details in the electronic supplementary material.
