# Supplementary material for: Cardiac myoglobin deficit has evolved repeatedly in teleost fishes
Source: Biol Lett. 2014 Jun;10(6):20140225. doi: 10.1098/rsbl.2014.0225 (PMC4090546; doi:10.1098/rsbl.2014.0225)
Supplement: Materials and methods [file rsbl-2014-0225-File004.pdf]

Electronic Supplementary Material

Reference numbering is as for the main document unless indicated as 'Supporting Ref'

Complete Materials and Methods

(a) Animal Sampling

Hearts were sampled from twenty-two Actinopterygian species. The source and number of individuals per species along with data on body size and sample usage is given in table S1. Fish were held in aquaria under normoxia at optimal habitat temperatures for 5d (Scottish Oceans Institute, University of St Andrews) before schedule-1 killing (UK home-office guidelines). Hearts were cut at the bulbus base, flushed with Ringers solution and digitally-photographed using a Leica Wild MC3 system (Carl Zeiss Microscopy). Hearts were flash-frozen in liquid nitrogen and stored at -80°C until RNA extraction.

(b) Databases and genomics

BLAST searches were performed against non-redundant protein and nucleotide NCBI (http://www.ncbi.nlm.nih.gov/) databases. The latest teleost genome assembly versions were compared in Ensembl (http://www.ensembl.org) using standard navigation tools and BLAST searches. All sequence alignment was performed using ClustalW [Supporting Ref 1] within Bioedit [Supporting Ref 2].

(c) *Mb* sequencing

Total RNA was extracted from the hearts of sixteen teleost species (table S1). Protocols used to extract RNA and determine its integrity, purity and concentration are described elsewhere [7]. 0.4-1.0 µg of RNA was reverse-transcribed and column-purified (QuantiTect and QIAquick kits respectively, Qiagen). Two degenerate primer pairs were designed from *Mb* sequences, the first designed to be

conserved in numerous species spanning the teleost phylogeny, and the second in numerous Acanthoptergian species specifically (table S2). These primer pairs were used in standard PCR reactions (30-40 cycles) employing first-strand cDNA templates from all species (individuals pooled when n>1). Successful PCR amplicons were cloned and sequenced by the Sanger method 6-12 times in both directions for nine species as described elsewhere [7] (accession numbers: *Pantodon buchholzi*: KJ561848; *Chitala chitala*: KJ561849; *Scleropages jardinii*: KJ561850; *Gnathonemus petersi*: KJ561851; *Trichopodus trichopterus*: KJ561852; *Amatitlania nigrofasciata*: KJ561853; *Apteronotus albifrons*: KJ561854; *Bunocephalus coracoideus*: KJ561855; *Anguilla Anguilla*: KJ561856).

(d) Selective pressure analyses

We estimated non-synonymous (d_N) and synonymous substitution (d_S) rates from pairwise species comparisons of *Mb* protein-coding sequences within Osteoglossiformes (*Pantodon buchholzi* vs. *Chitala chitala* vs. *Scleropages jardinii* vs. *Gnathonemus petersii*) and Acanthopterygii (*Trichopodus trichopterus* vs. *Amatitlania nigrofasciata* vs. *Tetraodon nigroviridis* vs. *Thunnus thynnus*). 130 of 147 possible codons were compared after primer regions were truncated. d_N/d_S was estimated in PAML [8] using a codon model [Supporting Ref 3].

(e) Quantitative PCR (qPCR)

SYBR®Green-based qPCR was performed on an MxPro3005p system (Agilent) for eleven teleost species (n=3/4) using species-specific primers (table S2) and a standardized input of heart cDNA. A detailed description of the qPCR reaction set-up, cycling conditions, estimation of assay efficiencies and data analysis is provided elsewhere [7]. As reference genes, we designed primers to conserved regions of 40S ribosomal protein S13 (*rps13*) and 40S ribosomal protein S29 (*rps29*) genes from species spanning the teleost phylogeny (table S2). *Mb*, *rps13* and *rps29* assays were run in triplicate within a plate for each species. Final mRNA level data (relative across species) was generated in

Genex v4.2.2 (MultiD Analyses AB) after correction for efficiency differences and normalization with *rps13* and *rps29*. All qPCR amplicons were cloned and sequenced as described above.

(f) Statistics

Statistics was performed in Minitab® v.16.1.0 (Minitab Inc.). We compared cardiac *Mb* mRNA levels in ten species (*G. aculeatus* excluded) using one-way ANOVA. Box-Cox transformation was used to ensure normality and homoscedasticity in the residuals. Tukey's test was used to establish differences among species (99.9% simultaneous confidence limit).

(g) Supporting References

Supporting Ref 1. Thompson JD, Higgins DG, Gibson TJ. 1994 CLUSTAL W: improving the sensitivity of progressive multiple sequence alignment through sequence weighting, position-specific gap penalties and weight matrix choice. *Nucleic Acids Res*. **22**, 4673-4680.

Supporting Ref 2. Hall TA. 1999 BioEdit: a user-friendly biological sequence alignment editor and analysis program for Windows 95/98/NT. *Nucleic Acids Symp Ser*. **41**, 95-98.

Supporting Ref 3. Goldman N, Yang Z. 1994 A codon-based model of nucleotide substitution for protein-coding DNA sequences. *Mol. Biol. Evol.* **11**, 725-736.
